# Efficacy of a High-Dose in Addition to Daily Low-Dose Vitamin A in Children Suffering from Severe Acute Malnutrition with Other Illnesses

**DOI:** 10.1371/journal.pone.0033112

**Published:** 2012-03-27

**Authors:** Samima Sattar, Tahmeed Ahmed, Choudhury Habibur Rasul, Debasish Saha, Mohammed Abdus Salam, Md Iqbal Hossain

**Affiliations:** 1 Clinical Sciences Division and Centre for Nutrition and Food Security, International Centre for Diarrhoeal Disease Research, Bangladesh (icddr,b), Dhaka, Bangladesh; 2 Department of Paediatrics, Khulna Medical College and Hospital, Khulna, Bangladesh; 3 Department of Social and Preventive Medicine, Centre for International Health, University of Otago, Dunedin, New Zealand; Aga Khan University, Pakistan

## Abstract

**Background:**

Efficacy of high-dose vitamin A (VA) in children suffering from severe acute malnutrition (SAM) has recently been questioned. This study compared the efficacy of a single high-dose (200,000 IU) in addition to daily low-dose (5000 IU) VA in the management of children suffering from SAM with diarrhea and/or acute lower respiratory tract infection (ALRI).

**Methods:**

In a randomized, double-blind, controlled clinical trial in icddr,b, Bangladesh during 2005–07, children aged 6–59 months with weight-for-height <−3 Z-score and/or bipedal edema (SAM) received either a high-dose VA or placebo on admission day. Both the groups received 5,000 IU/day VA in a multivitamins drop for 15 days and other standard treatment which is similar to WHO guidelines.

**Results:**

A total 260 children (130 in each group) were enrolled. All had diarrhea, 54% had concomitant ALRI, 50% had edema, 48.5% were girl with a mean±SD age of 16±10 months. None had clinical signs of VA deficiency. Mean±SD baseline serum retinol was 13.15±9.28 µg/dl, retinol binding protein was 1.27±0.95 mg/dl, and pre-albumin was 7.97±3.96 mg/dl. Median (inter quartile range) of C-reactive protein was 7.8 (2.1, 22.2) mg/L. Children of the two groups did not differ in any baseline characteristic. Over the 15 days treatment period resolution of diarrhea, ALRI, edema, anthropometric changes, and biochemical indicators of VA were similar between the groups. The high-dose VA supplementation in children with SAM did not show any adverse event.

**Conclusions:**

Efficacy of daily low-dose VA compared to an additional single high-dose was not observed to be better in the management of children suffering from SAM with other acute illnesses. A single high-dose VA may be given especially where the children with SAM may leave the hospital/treatment center early.

**Trial Registration:**

ClinicalTrials.gov NCT00388921

## Introduction

Diarrheal diseases, acute lower respiratory tract infection (ALRI), malnutrition, and vitamin A (VA) deficiency are common health problems in developing countries including Bangladesh. VA supplementation has proved effective in reducing childhood mortality in VA-deficient communities [1]. The World Health Organization (WHO) recommends giving high-doses of VA (200,000 IU for children more than one year old and half the dose to those aged 6–12 months) to children who suffer from severe protein-energy malnutrition (PEM) [2], [3], measles, prolonged diarrhea, and to VA deficient populations [4], [5]. There are quite a good number of studies those reflect positive effect of VA both in preventive as well as curative perspectives. However, some studies [6]–[11] that have examined the effect of high-dose VA supplementation on diarrhea and respiratory infections in children showed no beneficial effect, and in few studies, high-dose VA was associated with adverse effects, particularly in children with respiratory infection [9], [10], [12]–[15]. These varied responses of both beneficial and adverse effects are not yet fully understood. It has been argued that the deleterious effects were more frequent among well-nourished children particularly who had better VA status, but the role of nutritional status is difficult to estimate because most of the studies were done in non-severely malnourished or non SAM children.

Two clinical trials, performed by Donnen et al in Congolese [9] and Senegalese [16] children tested the effect of low-dose VA on all-cause morbidity. In the first study [9], effects of daily low-dose (5000 IU) and single high-dose (200,000 IU) VA were compared with a placebo. The duration and incidence of ALRIs were not found influenced by VA supplementation, regardless of the dose. However, ALRI on admission and in the course of hospitalization was rare in this sample of children and this fact could have masked some real effect. Neither a high-dose nor low-dose of VA had any significant effect on the duration of diarrhea and nosocomial diarrheaalso in the second study [16], which showed that nosocomial respiratory disease was lower in the low-dose group However, the children in both the studies were not exclusively with severely malnourished: mean weight-for-height (WHZ) of the studied children was -1.25 to -1.34 [9], and 44.7% to 48.6% had <−2 WHZ [16]; as well as few had edema: 18.2% to 21.3% [9] and 4.0% to 5.9% [16]. Interpretation of the studies [9], [16] in relation to the existing guidelines on VA supplementation in children with SAM was considered difficult, and this issue was flagged for further research in a meeting convened by WHO in September 2004 [17].

The purpose of the present study was to assess the efficacy of daily low-dose (5000 IU) versus an additional single high-dose (200,000 IU) on the recovery from diarrhea and ALRI including nosocomial infections in children with severe acute malnutrition (SAM).

## Methods

### Setting and Study Design

The protocol for this trial and supporting CONSORT checklist are available as supporting information; see Checklist S1 and Protocol S1. The study was conducted at the Dhaka Hospital of icddr,b which provides treatment to around 110,000 diarrheal patients with or without associated complications each year. The hospital is located in Dhaka, the capital city of Bangladesh with a population of about 15 million. The vast majority of the patients come from the poor socio-economic backgrounds from urban and peri-urban Dhaka, and about 6,000 of them, mostly under-five children, require hospitalization for five or more days in the longer stay ward for complications of diarrhea or presence of other infectious diseases, particularly ALRI, sepsis, and severe malnutrition.

This was a randomized, placebo-controlled double blind clinical trial conducted between June 2005 and May 2007. This study was approved by Research Review Committee and Ethical Review Committee of icddr,b. A written informed consent was obtained from the respective parents/guardians of the each participating children.

### Subject Recruitment

Hospitalized children aged 6–59 months of either sex were invited to be enrolled into the study if they fulfilled the following inclusion criteria: WHZ<−3 and/or bi-pedal nutritional edema i.e. they were suffering from SAM, and consent obtained from the guardian or parents. Children were excluded if they had clinically apparent congenital disorders that might affect growth, other acute or chronic diseases requiring continued hospitalization, active sign of VA deficiency or history of night blindness, active measles or history of measles with in the previous 8 weeks, received high-dose VA supplementation in the previous 3 months, and lack of a fixed address (to avoid difficulties in tracing for follow-up examinations).

### Randomization and Dosing

Eligible children were randomly assigned to either a high-dose or placebo using a computer-generated block randomization scheme with a block length of ten. Boh the groups received daily low-dose VA. Randomization was done by a researcher not involved in the study. A pharmaceutical company (Drug International limited, Bangladesh) prepared and supplied the high-dose VA capsules and placebo for the study. The placebo preparation and active drug were similar in consistency, color and taste. A person not affiliated with the study did the identification codes for the individual wrapped bottles of capsules. The bottles were unwrapped just before administration.

The research assistant screened the children/patients and then one of the investigators assessed the patients and they together took the consent to enroll the children in the study from the parents. Trained study personnel administered the study interventions (which were randomly pre-assigned to the children). The assignment of patients in the treatment groups were concealed to both the investigators and patients by enclosing the assignment card containing the code number for each patient into an opaque, sealed envelope which was opened only at the time of allocating patients to the study. At the start of trial, the high-dose group received an oral dose of 200,000 IU VA (100,000 IU VA if age<12 months) and the other (low-dose) group received placebo. Both the groups from the very first day received 5,000 IU/day VA in the form of multivitamin drops for 15 days. The date and time of administration was recorded. Each study day was defined as the 24 hours that started with the administration of the study drugs.

### Patient Management

The children were managed in two phases: (a) an initial stabilization phase and (b) the nutrition rehabilitation phase following the hospital's guidelines [18], which is very similar to the WHO guidelines [2], [3]. The initial treatment in the stabilization/acute phase began with admission to the hospital and lasted until the child's condition was stable and appetite had returned, which usually took 3–7 days. The principal tasks during initial treatment included treatment and prevention of hypoglycemia, hypothermia, dehydration, electrolyte imbalance, shock, frequent feeding with a milk-based diet, and treatment of infections and other problems. This phase of treatment ended when the child got back a normal appetite. Nutritional rehabilitation was done as per the standard practice of the hospital. This included giving rice-lentil-based foods [19] (*khichuri*: a cooked mix of rice, lentils, vegetables and soybean oil and *halwa*: a sweet thick porridge made from cereal powder and lentils) and a high energy milk-based diet which was gradually tapered in amount. Breastfeeding was encouraged and continued between formula feeds. Iron, which was not given in the initial phase, was started during the nutrition rehabilitation phase at a dose of 3 mg of elemental iron/kg per day. The other tasks during nutritional rehabilitation phase were: encouraging the child to eat the offered food stimulating emotional and physical development, and preparing the mother or caretaker to continue to look after the child at home following discharge. A trained health worker counseled the mother/caregiver in the following areas: (a) breastfeeding and preparation of nutritious solid food with available food ingredients; (b) management of fever and diarrhea at home; (c) danger signs of common illnesses; (d) benefits of immunization. The discharge criteria were: absence of sign of infection, general condition had improved, and the children had achieved an edema free WHZ of >−2 or having stayed in the nutritional rehabilitation unit for 15 days (which ever was longer).

### Data Collection

An interviewer-administered pretested questionnaire was used to collect information on socioeconomic and dietary data, obtained from the mothers or guardians after the children had entered the trial. During hospitalization, one of the investigators assessed the study children at least once daily and recorded morbidity including weight data on a pre-designed and pre-tested case report form (CRF). Length (for less than 2 years old), height (for 2 to 5 years old), mid-upper-arm-circumference (MUAC) and occipito-frontal (head) circumference were measured at base line, on day 7 and on day 15. Morbidity data including number and consistency of stool, vomiting, fever (rectal temperature) were measured thrice daily in every 8 hours interval, and all other symptoms over the preceding 24 hours. Each child was weighed every morning, without clothes to the nearest 10 gm using an electronic weighing scale (model 6810: Seca Corporation, Columbia, MD), which was calibrated daily. Length and height were measured with a locally made standard length/height board to the nearest 0.1 centimeter. MUAC (measured half way between the elbow and the shoulder of the left arm) and head circumference were measured with a nonexpanding plastic tape and recorded to the nearest 01 mm. The results are reported in relation to the new (2006) WHO growth standards and expressed as WHZ, weight-for-age (WAZ) and height-for-age (HAZ) z scores.

Blood samples were taken from anticubital venipuncture for measurement of electrolytes, creatinine, complete blood count, total protein, albumin, retinol, retinol binding protein (RBP), glucose, pre-albumin and C-reactive protein (CRP) on the admission day before the administration of high-dose VA or placebo. Routine and microscopic examinations of stool and urine and ELISA of stool sample (to detect Rota virus), x-ray chest, pulse oximetry and other tests including blood culture were done as the patient's condition demanded. Serum retinol and RBP were again measured on study days 3 and 15.

### Laboratory Procedures

Serum retinol was measured by high-pressure liquid chromatography, a method adapted from that of Vanderpas and Vertongen [20]. RBP was measured with a single-radial immune diffusion technique using commercially available plates (The Binding site, Birmingham, United Kingdom). Ring diameters (in mm) were read with an electronic single-radial immune diffusion plate reader 72 hours after inoculation of serum sample. For each plate, 3 wells were used for standards of different concentrations and 1 for quality control [21]. Other laboratory measures/assays were done with standard procedures.

### Outcome Variables and Definitions

The primary outcome measures were clinical success- defined as cessation of diarrhea and improvement of ALRI (no temperature, fast breathing, and rales on chest auscultation) within 48 hours of study drug administration. The secondary outcome variables were any adverse event or clinical features of VA toxicity (bulging fontanel, headache, vomiting, seizure, change in mental condition), changes serum retinol and RBP levels, duration of resolution of diarrhea, ALRI, edema, dermatosis, and other illness (if any), changes in weight and length/height, nosocomial morbidities, and mortality. Diarrhea was defined as passage of 3 or more watery or semi liquid stools in a 24 hours period. The number of passage of stools per 8 hours was recorded. An episode of ALRI was defined as presence of at least 3 of the following i) cough, ii) fever, iii) rales, iv) increased age-specific respiratory rates (>50 breaths and >40 breaths per minute in 6 to 11 months and 12 to 59 months old group of children respectively) and v) chest x-ray findings suggestive of pneumonia. An episode was considered elapsed when there was 48 hours without any signs or symptoms of that specific illness. An episode was considered new or a nosocomial infection if it was preceded by an interval of 48 hours without clinical features of that illness or if an infection newly appeared 48 hours after hospitalization.

### Sample Size

In the Dhaka Hospital of icddr,b about 60% of the severely malnourished children has cessation of diarrheal illness and improvement of ALRI by 48 hours (unpublished data, ICDDR,B, 2005). Sample size estimation per group was done considering the above mentioned two primary outcome variables. Considering 80% power (1-beta) and 0.05 significance level (type I error) and ∼12% dropout rate, 130 children in each group would be able to detect 17% or more difference in primary outcome variables between the groups, and we enrolled a total of 260 children with severe acute malnutrition in the two treatment groups.

### Data Quality Control, Processing and Analysis

Strict data quality control was maintained throughout the trial. Each case record form was reviewed, first by a research assistant and then by the principle or co-principle investigator before being handed over for data entry. Data were entered into a computer by research assistant, and 10% of the entered data was checked by one of the investigators. In addition, data were printed out periodically for visual checks. Data were entered and analyzed using SPSS for Windows (version 11.5; SPSS Inc., Chicago, IL, USA). For normally distributed continuous variables, means were compared using unpaired t-tests after checking the equality of variance (Levene's test). For non-normally distributed continuous variables, the Mann-Whitney test was performed. Differences in proportions were compared by the Chi-square test or Fisher's exact test if the expected number in any cell was <5. A probability of less than 0.05 was considered statistically significant. Time taken to resolution of diarrhea, ALRI, edema and other illnesses were compared between the groups by survival analysis. Logistic regression was used to analyze incidence of nosocomial morbidities, where in addition to treatment groups, admission retinol and CRP levels, and edema and stunting status were included in the model. All these variables were transformed into dummies and were coded as either 0 (to indicate the reference category) or 1 (to indicate the category at risk). Adjusted odds ratios and their 95% confidence intervals were derived from the final logistic model.

## Results

**Figure 1:**
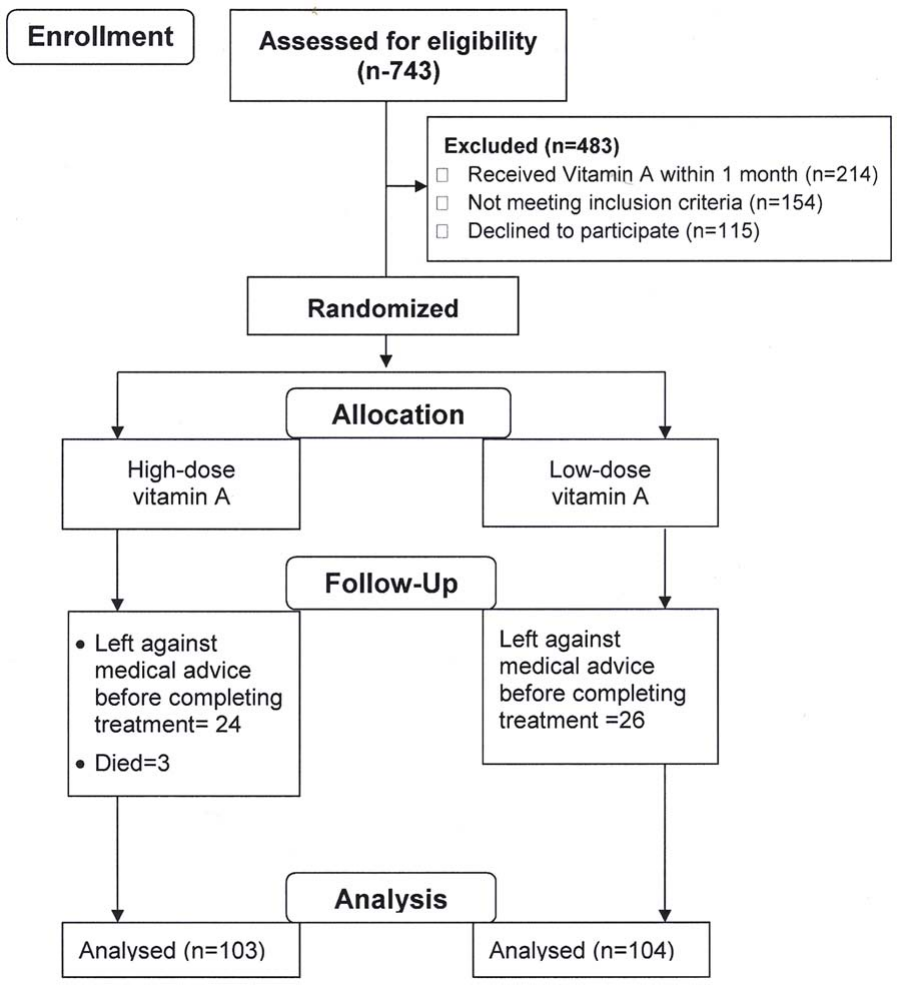
Study profile.

We assessed 743 children and 483 were excluded and the remaining 260 children were randomized (Figure 1). Of them 48.5% were girls, 50% had edema, 71% were stunted (length or height-for-age <−2 Z score), all had diarrhea, 54% had concomitant ALRI and none had clinical signs of VA deficiency. Their mean ± SD baseline age was 16.2±9.9 months, serum retinol was 13.15±9.28 µg/dl, RBP was 1.27±0.95 mg/dl, and pre-albumin was 7.97±3.96 mg/dl. Median (inter quartile range) of CRP was 7.8 (2.1, 22.2) mg/L, and 45% had ≥10 mg/L. Of the enrolled children 42% were VA deficient (serum retinol <10 µg/dl), 39% had low VA (serum retinol between 10–20 µg/dl), and only 19% had >20 µg/dl serum retinol (dectected after enrollment in admission blood sample). Children of the two groups did not differ at baseline in any of the nutritional, demographic, socio-economic, and illnesses/admission morbidities (Table 1). Over the first 48 hours percent of children who had resolution of watery diarrhea, invasive diarrhea and ALRI (primary outcome variables), and over total 15 days treatment period time taken for cure/resolution of admission morbidities were similar between the groups (Table 2), as well as between the edematous and non-edematous children, whose baseline serum retinol and other biochemical characteristics were also similar (data not shown). Survival analysis also revealed no difference in resolution times of different morbidities between the two treatment groups (data not shown). Similarly over the first 48 hours and 15 days treatment period changes in serum retinol, RBP were similar between the groups (Table 3). Table 4 shows that changes of different anthropometric measures were comparable between the high-and low-dose VA treatment groups. In further exploratory analyses by logistic regression, considering the treatment groups, initial retinol and CRP levels, and admission edema and stunting status in the model, it was found that incidence of nosocomial cough was 39% less likely in high-dose VA group, adjusted (a) OR: 0.61, P = 0.056, and nosocomial ALRI was more in stunted children, aOR: 2.96, P = 0.002 (Table 5).

**Table 1:** Baseline characteristics of children by treatment group.

	High Dose Vitamin A Group(N = 130)	Low Dose Vitamin A Group(N = 130)
Age (mo): mean ± SD	15.7±8.9	16.8±10.9
Female	66 (51)	60 (46)
Predominant breast feeding up to first 6 months	84 (65)	96 (74)
Length: mean ± SD	69.7±6.8	70.3±8.7
Length or height-for-age Z score*: mean ± SD	−2.72±1.27	−2.74±1.24
*Among non edematous children*		
Weight (kg): mean ± SD	6.40±1.34	6.84±1.64
Weight-for-age Z score*: mean ± SD	−4.16±0.77	−4.08±0.77
Weight-for-length or height Z score*: mean ± SD	−3.32±0.53	−3.30±0.32
Children admitted with edema	66 (51)	64 (49)
MUAC† (mm): mean ± SD	111.2±10.7	111.6±11.3
Head circumference (cm): mean ± SD	42.1±2.2)	42.0±2.6
BCG‡	120 (92)	117 (90)
Diphtheria, tetanus, pertussis and oral polio		
(3^rd^ dose completed)	122 (94)	115 (88)
Measles	54 (59)	50 (56)
Income (taka per month) (1 US $ = 65 taka)		
>10,000	2 (1.5)	(0)
3,000–10,000	27 (20.8)	29 (22.3)
<3,000	101 (77.7)	101 (77.7)
Admitted with acute watery diarrhea	117 (90)	118 (91)
Admitted with invasive diarrhea	13 (10.0)	12 (9)
Admitted with fever	72 (55)	65 (50)
Admitted with cough	73 (56)	66 (51)
Admitted with pneumonia/ALRI§	74 (57)	67 (52)
Admitted with skin change/dermatosis	16 (12)	14 (11)
Serum retinol (µg/dl): mean ± SD	13.3±9.1	13.0±9.5
<10	57 (44)	53 (41)
10–20	46 (35)	54 (41)
>20	27 (21)	23 (18)
RBP¶ (mg/dl): mean ± SD	1.26±0.94	1.29±0.95
CRP∥ (mg/L): median (inter quartile range)	9.7 (2.4, 23.4)	7.5 (1.9, 20.2)
Pre-albumin (mg/dl): mean ± SD	7.64±3.95	8.31±3.95
Hematocrit (%): mean ± SD	32.7±3.8	32.7±4.5
Serum total protein (g/L): mean ± SD	67.1±10.4	67.7±11.6
Serum albumin (g/L): mean ± SD	37.6±7.0	38.0±7.4

Data are n (%) unless otherwise indicated.

* WHO new (2006) growth standard;

† MUAC: Mid upper arm circumference;

‡ BCG: Bacille Calmette Guerin.

§ ALRI: Acute lower respiratory tract infection;

¶ RBP: Retinol binding protein;

∥ CRP: C-reactive protein.

No significant difference is observed for any variable between the groups.

**Table 2:** Resolution of morbidities by treatment group.

	High Dose Vitamin A Group	Low Dose Vitamin A Group	P Value
*Resolution/improvement by 48 hours: n (%) [primary outcome variables]*			
Resolution of acute watery diarrhea	51 (44) [117]	52 (44) [118]	0.314
Resolution of dysentery	7 (54) [13]	8 (67) [12]	0.790
Improvement of ALRI*			
Cough	42 (58) [73]	35 (53) [66]	0.723
Fever	52 (72) [72]	48 (74) [65]	0.445
Rales	7 (44) [16]	6 (50) [12]	0.541
*Duration of morbidities: mean ± SD [primary outcome variables]*			
Acute watery diarrhea (hours)	73.9±42.3	64.6±40.8	0.071
Invasive diarrhea (hours)	97.8±66.9	65.5±29.5	0.360
Cough (day)	3.15±2.56	2.85±1.92	0.924
Fever (day)	2.00±1.51	1.48±0.98	0.288
Rales on chest auscultation (day)	4.69±4.64	3.86±1.95	0.635
Edema (day)	7.11±4.08	7.58±3.33	0.199
Skin change (day)	4.80±2.11	4.18±2.79	0.304

[n]: number of subject for that specific variable.

* ALRI: Acute lower respiratory tract infection.

**Table 3:** Changes of serum retinol and retinol binding protein (RBP) over time by treatment group.

	High Dose Vitamin A Group(N = 127)	Low Dose Vitamin A Group(N = 126)	P Value
S. Retinol (µg/dl) on day 3: mean ± SD	24.1±11.4	23.7±11.4	
<10	10 (7.9)	15 (11.9)	
10–20	39 (30.7)	30 (23.8)	
>20	78 (61.4)	81 (64.3)	0.822
Difference (day 3 - baseline) of S. Retinol (µg/dl) after 48 hours: mean ± SD	10.8±11.5	10.8±11.7	
median (inter quartile range)	9.8 (4.5, 16.5)	9.9 (4.7, 5.7)	0.958
RBP* (mg/dl) on day 3: mean ± SD	2.30±1.12	2.28±1.13	0.926
Difference (day 3 - baseline) of RBP (mg/dl) after 48 hours: mean ± SD	1.04±1.16	1.00±1.18	
median (inter quartile range)	0.92 (0.24, 1.71)	1.01 (0.21, 1.58)	0.904
	(N = 103)	(N = 104)	
S. Retinol (µg/dl) on day 15: mean ± SD	40.4±11.6	39.8±11.6	
<10	0	0	
10–20	0	3 (3)	0.718
>20	103 (100)	101 (97)	
Difference (day15 - baseline) of S. Retinol (µg/dl) after 14 days: mean ± SD	27.2±13.8	27.3±14.1	0.979
RBP (mg/dl) on day 15: mean ± SD	3.82±1.08	3.81±1.09	0.920
Difference (day 15 - baseline) of RBP (mg/dl) after 14 days: mean ± SD	2.58±1.33	2.57±1.35	0.970

Data are n (%) unless otherwise mentioned.

* RBP: Retinol binding protein.

**Table 4:** Change in nutritional status by treatment group.

Changes over 2 weeks(mean ± SD)	High dose vitamin A group(N = 103)	Low dose vitamin A group(N = 104)	P Value
*Weight (kg)*			
On day 15	6.75±1.44	6.71±1.91	0.862
Difference from admission day	0.74±0.43	0.69±0.51	1.000
*Length (cm)*			
On day 15	69.8±6.8	69.8±9.0	0.994
Difference from admission day	0.2±0.3	0.1±0.3	0.415
MUAC* (cm)			
On day 15	117 (±11)	116±12	0.510
Difference from admission day	6.0±4.2	5.2±5.0	0.298
*Head circumference (cm)*			
On day15	42.5±2.1	42.2±2.7	0.368
Difference from admission day	0.39±0.37	0.37±0.35	0.788

**Table 5:** Results of exploratory analyses by logistic regression on occurrence of nosocomial morbidities after adjusting the other co-morbidities/conditions.

Nosocomial morbidity	Adjusted OR	95.0% C.I. of adjusted OR		P Value
		Lower	Upper	
**ALRI** † **(all children):** variables in model: treatment group, admission retinol, CRP, edema and stunting status				
*High dose vitamin A group*	0.63	0.36	1.09	0.099
*Admission retinol <20 µg/dl*	0.70	0.35	1.40	0.312
*Admission CRP >10 mg/L*	0.74	0.42	1.31	0.295
*Edematous children*	1.12	0.64	1.94	0.696
*Stunted children*	2.96	1.47	5.96	0.002
**Diarrhea(all children):** variables in model: treatment group, admission retinol, CRP, edema and stunting status				
*High dose vitamin A group*	1.25	0.67	2.34	0.483
*Admission retinol <20 µg/dl*	1.65	0.68	4.00	0.269
*Admission CRP >10 mg/L*	0.88	0.46	1.66	0.690
*Edematous children*	1.21	0.65	2.26	0.541
*Stunted children*	1.01	0.50	2.01	0.986

CRP: C-reactive protein;

† ALRI: Acute lower respiratory tract infection.

The number (%) of children left against medical advice before completing the minimum 15 days treatment were 24 (19%) and 26 (20%) in high- and low-dose group respectively (figure 1). No significant characteristic was found which could predict or help to identify them from the children who completed the full study periods. A total three children died, and all received high-dose VA, but all were critically ill and had sepsis/septicemia. The first one was a 13 months old girl with marasmic kwashiorkor, diarrhea suppurative otitis media, sepsis, thrush and hypokalemia. The second one was a 15 months old boy with severe wasting, Salmonella group B enteritis with thrush and hypokalemia, and the third one was a boy with marasmic kwashiorkor, diarrhea, Klebsiella septicemia, dermatosis and hypokalemia. The mean ±SD serum retinol of the children who died was 11.90±7.76 µg/dl, and the deaths were not considered to be related to VA doses.

## Discussion

This randomized, double-blind, placebo-controlled clinical trial showed that the efficacy of low-dose VA supplementation was not found to be better compared to the current practice of an initial high-dose VA supplementation in reducing morbidity in Bangladeshi children with severe acute malnutrition (SAM) suffering from diarrhea and/or ALRI, who were invariably VA deficient. The major strengths of our trial were the rigorous study design and the careful standardization of the clinical assessments. Randomization and the double-blind design minimized the possibility of confounding or differential misclassification. To our knowledge, this was the first clinical trial to compare the effect of daily low-dose, and additional high-dose VA supplementation on all major and common morbidities in exclusively SAM children.

In Bangladesh, high potency (200,000 IU for 1–5 years old and 100,000 IU for 6–11 months old) VA is routinely supplemented to under-five children every 6 months. In severely malnourished children, nutrient deficiencies including that of protein, fat and zinc could be limiting factors for the absorption and utilization of the lipid-soluble vitamin [22], [23]. A significant proportion of supplemented VA may be excreted in faces and urine, and urinary excretion is substantially increased in acute infections including diarrhea that malnourished children frequently suffer from. It is estimated that malnourished children lose 0.1 µmol (97 IU) of retinol/day through urine during acute illness [21]. Children with protein deficiency and edema, reduced their RBP synthesis [24], which is synthesized in the liver and is required for carrying retinol to the target organs and tissues. Acute infections are also associated with acute phase response and a transient decrease in serum retinol level [25]. This is due to reduced transcription of messenger RNA for RBP, resulting in decreased release of RBP from the liver into the blood [26], [27]. It is likely that liver utilizes all of its available resources for rapid synthesis of proteins that are required for the host defense in acute infection, thereby limiting the synthesis of RBP.

It seems unlikely that the high-dose VA could have induced adverse effects in this malnourished and VA deficient children of our study, because most of the children were VA deficient on the basis of their serum retinol concentrations (only 19% had an admission serum retinol level >20 µg/dl), with a mean ±SD serum retinol concentration of 13.15±9.28, which was similar in edematous (13.1±9.9 µg/dl) and non-edematous (13.2±8.6 µg/dl) children. Furthermore, the increases in serum retinol concentrations after 48 hours as well as on day 15 were similar in high- and low-dose groups. Similar phenomenon was also observed in Congolese study [9], where the day 7 serum retinol was observed comparable (p = NS) in the 3 groups of children who received high- or low-dose or a placebo and the changes were 0.20±0.32, 0.19±0.36 and 0.17±0.37 µmol/L respectively. In anaother study^7^ done among the apparently well nourished children (WHZ>−1) it was seen that among those with a baseline serum retinol was <0.70 µmol/L (equivalent to <20 µg/dl) the mean changes in serum retional over 7 days were found similar between VA suplemented vs. no supplemented group, 0.67 vs. 0.73 µmol/L respectively (p = NS).

Till now the understanding of the molecular mechanisms by which protein status affects VA dependent functions remains limited. Compared with healthy children, protein deficient children and edematous children synthesize less RBP and release less RBP from the liver, and thus have lower RBP concentrations [24]. Furthermore, protein deficiency possibly interferes with the liver storage of VA, as it does in rats [28]. Thus, in protein deficient children, the response to a massive dose of VA could be blunt if only checked by biochemical parameters. We do not believe that the children in our study had received an excess of VA. Mortality in all three children was in high-dose group in children but the causes of deaths were very much clear, and perhaps not related to the doses of VA.

Our findings are consistent with the results of the trial by Henning et al [29] in Bangladesh, where they found no significant difference in the duration of diarrhea and total stool output between children hospitalized with non-cholera acute diarrhea receiving 200,000 IU VA and children receiving a placebo. Coutsoudis et al [30] found no significant differences in the severity of diarrhea episodes at 6-week and 6-month follow-ups of a group of African children who received 200,000 IU VA on admission, on days 2 and 8, and at 6 weeks nor in the placebo group. Most population-based studies of VA that were conducted outside of hospitals failed to find any significant differences in the incidence of mild diarrhea (3 or 4 loose stools a day) between VA supplemented and placebo groups [30]–[34] In a population-based, placebo-controlled study in India, weekly low-doses (2500 mg) of VA for 1 year did not influence the incidence, severity, or duration of diarrhea or respiratory infections in malnourished children [32]. In Guatemala, high-dose VA supplementation failed to show any benefit in children with radiographically confirmed ALRIs [7].

On the other hand, in South Africa, children hospitalized with acute measles recovered more rapidly from pneumonia [30], [35], [36] and the frequency and severity of pneumonia episodes were significantly lower in the VA–supplemented group [30]. Recent work suggests marked improvements in immune responses after VA supplementation [37]. Julien et al [38] in a randomized, double-blind, placebo-controlled clinical trial in Mozambican children hospitalized with non-measles ALRIs reported beneficial effect of high-dose of VA. Bangladeshi children with shigellosis who received VA were less likely to be clinically ill by day 5 than were children given placebo [39]. In India, children who had pre-existing VA deficiency and PEM had a significant reduction in the duration of diarrhea who received high-dose VA supplements [40] Vietnamese moderately malnourished VA-supplemented children had a significantly shorter time of hospitalization [41]. Sommer at al [42] found a decrease in mortality by 34% in 450 villages in northern Sumatra in preschool children who received 6 monthly supplementation of 200,000 IU VA compared to the control children. A more recent study had observed supplementation of vitamin A to under-five children to be associated with decreased frequency of diarrhea, ALRI and subsequent malnutrition [43].

WHO recommends single high-dose of VA supplementation for severely malnourished children [2], [3]. The recommendation is based on studies in communities that showed benefit of the cheapest intervention, which is quicker to implement into health programs. Repeated low dosing for longer time often may not possible in situation where the malnourished children leave the hospital/treatment centers early due to mothers'/guardians' other responsibilities. We conclude that efficacy of daily low-dose (5,000) VA compared to an additional single high-dose (200,000 IU for 1–5 years old and 100,000 IU for 6–11 months old) was not observed to be better in the management of children suffering from SAM with diarrhea and/or other acute illnesses. Morover the high-dose VA supplementation in children with SAM did not show any advers event. Thus single high-dose VA may be given safely to augment the hepatic reserves and especially where the children with SAM may leave the hospital/treatment center early.

## Supporting Information

Checklist S1 **CONSORT Checklist.**
(DOC)

Protocol S1 **Trial Protocol.**
(DOC)
