# Supplementary material for: Efficacy of a High-Dose in Addition to Daily Low-Dose Vitamin A in Children Suffering from Severe Acute Malnutrition with Other Illnesses
Source: PLoS One. 2012 Mar 27;7(3):e33112. doi: 10.1371/journal.pone.0033112 (PMC3314008; doi:10.1371/journal.pone.0033112)
Supplement: Protocol S1 — Trial Protocol. (DOC) [file pone.0033112.s002.doc]

**Trial Protocol**

**Project Title**: The effect of 2,00,000 IU of vitamin A followed by 5,000 IU daily doses versus daily 5,000 IU of vitamin A on recovery from diarrhoea and acute lower respiratory infections in severely malnourished hospitalized children.

**Short protocol title (in 50 characters including space):** High and low dose vitamin A in severely malnourished children with diarrhoea and ALRI.

**Theme: *(Check all that apply)***

Nutrition  Environmental Health

Emerging and Re-emerging Infectious Diseases  Health Services

Population Dynamics  Child Health

Reproductive Health  Clinical Case Management

Vaccine evaluation  Social and Behavioural Sciences

HIV/AIDS

### Key words: Vitamin A, Children, Severe malnutrition, Retinol, Retinol binding protein, Diarrhoea, ALRI

**Relevance of the protocol:**

Vitamin A deficiency is an important global health problem including Bangladesh. The problem is greater among under-five children, particularly those malnourished. Vitamin A supplementation reduces morbidity from diarrhoeal diseases and also prevents diarrhoea episodes. On the other hand, there are conflicting reports on the role of vitamin A supplementation on morbidity from acute lower respiratory infections (ALRI) including pneumonia; in non-malnourished children supplementation has actually been reported to be associated with increased ALRI incidence and morbidity. The WHO committee (Underwood et al, 1992) has reviewed both the risk and benefit of mega dose (200,000 IU) vitamin A supplementation during acute illness, particularly diarrhoea, irrespective of the nutritional status of under-5 children (IVACG Policy Statement on Vitamin A, Diarrhoea, and Measles. <http://www.ilsi.org/measles.html> at 1500h on 13 July 2005). In Bangladesh high potency (200,000 IU) of vitamin A is routinely supplemented to under-5 children every 6 months. Absorption of vitamin A precursors from the GI tract is reduced in severely malnourished children, who also lack in retinol binding protein (RBP), required for transportation of retinol to target organs. It can thus be assumed that a significant proportion of supplemented vitamin A is excreted in feces and urine of malnourished children. The excretion of vitamin A substantially increases during acute infections including diarrhoeal diseases. On the other hand, free vitamin A, consequence of reduced RBP, increases their concentration in the body increasing the possibility of adverse events including “pseudotumor cerebri”. It has recently been observed that low-dose daily supplementation of vitamin A to malnourished children produces comparable or better effect on recovery from acute illness and also in preventing infectious diseases among under-five children. However, the studies have limitations such as small sample size, delayed assessment of retinol after supplementation, which needs to be addressed in a well-designed clinical trial. We hope that our proposed study will enable us to compare the efficacy of low-dose daily administration of vitamin A with that of initial mega dose followed by daily low dose of vitamin in children presenting with acute diarrhoeal diseases with or without ALRI. If the results of this study indicate that the daily low-dose has similar efficacy to that of the currently recommended mega dose followed by daily dose of vitamin A that would have important programmatic implications.

**Centre’s priority: (as per strategic plan)** Nutrition. Code No. 3

**Programmes**

Child Health Programme  Health and Family Planning Systems Programme

Nutrition Programme  Population Programme

Programme on Infectious Diseases & Vaccine Science  Reproductive Health Programme

Poverty and Health Programme  HIV/AIDS Programme

**Population: Inclusion of special groups** (*Check all that apply)*:

Gender  Pregnant Women

Male  Fetuses

Female  Prisoners

Age  Destitutes

0 – 5 years  Service providers

5 – 9 years  Cognitively Impaired

10 – 19 years  CSW

20 – 64 years  Others (specify _______________________)

65 +  Animal

**NOTE:** It is the policy of the Centre to include men, women and children in all biomedical and behavioural research projects involving human subjects unless a clear and compelling rationale and justification (e.g. gender specific or inappropriate with respect to the purpose of the research) is there. Justification be provided in case inclusiveness of study participants is not proposed in the study.

**Project / study Site (***Check all the apply)*:

Dhaka Hospital  Mirsarai

Matlab Hospital  Patyia

Matlab DSS area  Other areas in Bangladesh _______________

Matlab non-DSS area  Outside Bangladesh

Mirzapur name of country: ______________________

Dhaka Community  Multi centre trial

Chakaria (Name other countries involved)

Abhoynagar  **______________________________________**

**Type of Study** *(Check all that apply)*:

Case Control study  Cross sectional survey

Community based trial / intervention  Longitudinal Study (cohort or follow-up)

Program Project (Umbrella)  Record Review

Secondary Data Analysis  Prophylactic trial

Clinical Trial (Hospital/Clinic)  Surveillance / monitoring

Family follow-up study  Others

**NOTE:** All clinical studies/trials should be registered in appropriate websites, preferably at [www.clinicaltrials.gov](http://www.clinicaltrials.gov/). When the study is registered in website(s), the PI should provide website address, registration number, and date of registration to the Committee Coordination Secretariat for entering these information into the Centre’s database of your research protocol.

**Targeted Population** *(Check all that apply)*:

No ethnic selection (Bangladeshi)  Expatriates

Bangalee  Immigrants

Tribal groups  Refugee

**Consent Process** *(Check all that apply)*:

Written  Bengali language

Oral  English language

None

**Proposed Sample size: 260** Total sample size: 260

Sub-group: High dose vitamin A-130 Low-dose vitamin A: 130

_______________________________________ ______________________________________

**Determination of Risk: Does the Research Involve** *(Check all that apply)*:

Human exposure to radioactive agents?  Human exposure to infectious agents?

Fetal tissue or abortus?  Investigational new drug

Investigational new device?  Existing data available via public archives/source

(specify________________________)  Pathological or diagnostic clinical specimen only

Existing data available from Co-investigator  Observation of public behavior

New treatment regime

## Yes/No

Is the information recorded in such a manner that subjects can be identified from information provided directly or through identifiers linked to the subjects?

Does the research deal with sensitive aspects of the subject's behaviour; sexual behaviour, alcohol use or illegal conduct such as drug use?

Could the information recorded about the individual if it became known outside of the research:

a. place the subject at risk of criminal or civil liability?

b. damage the subject's financial standing, reputation or employability; social rejection, lead to stigma, divorce etc.

**Do you consider this research** *(Check one)*:

greater than minimal risk  no more than minimal risk

only part of the diagnostic test

Minimal Risk is "a risk where the probability and magnitude of harm or discomfort anticipated in the proposed research are not greater in and of themselves than those ordinarily encountered in daily life or during the performance of routine physical, psychological examinations or tests. For example, the risk of drawing a small amount of blood from a healthy individual for research purposes is no greater than the risk of doing so as a part of routine physical examination".

**PROJECT SUMMARY:** Describe in concise terms, the hypothesis, objectives, and the relevant background of the project. Describe concisely the experimental design and research methods for achieving the objectives. This description will serve as a succinct and precise and accurate description of the proposed research is required. This summary must be understandable and interpretable when removed from the main application. **( TYPE TEXT WITHIN THE SPACE PROVIDED).**

Vitamin A deficiency is a major nutritional problem in many developing countries, including Bangladesh. Globally, an estimated 140-250 million under-five children are deficient of vitamin A and 5 million of them have severe deficiency. Like other micronutrients, malnourished children are also deficient of vitamin A, and frequent common illnesses e.g. diarrhoea, and upper respiratory tract infections including pneumonia in this age group worsens their deficiency states.

The low serum retinol levels in malnourished children perhaps represents a combined effect of lower absorption of vitamin A precursors from their intestine and lack of carrier protein, retinol-binding protein (RBP), which is synthesized in liver and required for carrying retinol to the target organs, leading to development of the features of vitamin A deficiency. In infectious diseases including diarrhoea, excretion of vitamin A is substantially increased in urine and its functions (e.g. integrity of epithelial cells throughout the body)[1] are seriously compromised at tissue levels.

Acute lower respiratory tract infections (ALRI) including pneumonia, and diarrhoeal diseases are the two commonest diseases that affect under-five children. The beneficial role of high-dose vitamin A supplementation, as an adjunct to treatment of diarrhoea, has been documented in several studies. However, high dose vitamin A does not provide any benefit in the management of ALRI in well-nourished children with adequate vitamin A stores, and in fact, has been associated with increased incidence of ALRI. Some studies have observed increased incidence of diarrhoea and symptomatic respiratory infections after 2 weeks of receiving high dose of vitamin A. One study failed to observe any difference in the mortality (8% vs. 8.4%) and duration of hospitalization (10 days vs. 9 days) between groups of children receiving a high and a low dose of vitamin A. In a subgroup analysis, mortality was lesser in the group of children who received daily low dose compared to those who received single high dose (9.7% vs. 11.1%), and the reduction in deaths was more pronounced in children who had oedematous malnutrition on admission. Results of these two studies, indicating higher deaths in moderately malnourished and oedematous children receiving high dose vitamin A, need to be further examined in a well-designed study because of its implications on vitamin A supplementation programmes.

With the above background, we propose a study to examine the effects of daily low dose vitamin A (5000 IU for 15 days) in severely malnourished children, aged 6-59 months, with diarrhoea, with or without pneumonia, and compare that with initial high dose (200,000 IU) followed by a daily low doses (5000 IU for 14 days) of vitamin A in a randomized, controlled clinical trial. All children enrolled in the study would receive the standard care and treatment for their diarrhoea and associated illnesses. Serum concentrations of retinol and retinol binding protein will be measured in study children before randomization, after 48 hours (i.e. on 3^rd^ day) and 14 days later (i.e. on 15^th^ day). The duration of diarrhoea, and proportion of children recovering from pneumonia (return of respiratory rate to normal, and resolution of fever, and ability to eat and drink well) within 48 hours will be the primary outcome measures, and the findings would be correlated with serum retinol and retinol binding protein. We hope that this study might provide valuable information in defining an evidence-based programme for vitamin A supplementation to severely malnourished children.

DESCRIPTION OF THE RESEARCH PROJECT

**Hypothesis to be tested:**

Concisely list in order, in the space provided, the hypothesis to be tested and the Specific Aims of the proposed study. Provide the scientific basis of the hypothesis, critically examining the observations leading to the formulation of the hypothesis.

We hypothesize that a daily low dose (5,000 IU) administration of vitamin A will be more effective than a single high dose (2000,000 IU) followed by daily low doses in the recovery of diarrhoea and pneumonia in severely malnourished hospitalized children, and that it will be safe to use this low-dose schedule.

**Specific Aims:**

Describe the specific aims of the proposed study. State the specific parameters, biological functions/ rates/ processes that will be assessed by specific methods **(TYPE WITHIN LIMITS).**

Primary aim

To compare the proportion of severely malnourished, hospitalized children, aged 5-59 months, experiencing resolution of diarrhoea and ALRI/pneumonia in association with a single mega dose (200,000 IU) followed by daily low dose (5,000 IU) of vitamin versus daily low dose (5,000 IU) of vitamin A as an adjunct to standard management of their primary illnesses.

Secondary aims: The secondary aims of the study are to compare:

1. Duration of hospital stay, and the rates of weight gain more than 10 gm/kg/day among children in the two intervention groups.
2. Case-fatality among children in the two intervention groups, and
3. Serum retinol and retinol binding protein (RBP) levels on admission, day 3 and on day 15.

**Background of the Project including Preliminary Observations**

Describe the relevant background of the proposed study. Discuss the previous related works on the subject by citing specific references. Describe logically how the present hypothesis is supported by the relevant background observations including any preliminary results that may be available. Critically analyze available knowledge in the field of the proposed study and discuss the questions and gaps in the knowledge that need to be fulfilled to achieve the proposed goals. Provide scientific validity of the hypothesis on the basis of background information. If there is no sufficient information on the subject, indicate the need to develop new knowledge. Also include the **significance and rationale** of the proposed work by specifically discussing how these accomplishments will bring benefit to human health in relation to biomedical, social, and environmental perspectives. **(DO NOT EXCEED 5 PAGES, USE CONTINUATION SHEETS).**

An estimated 140–250 million under-five children are deficient in vitamin A globally, which is associated with increased mortality and morbidity. A relationship between the severity of vitamin A deficiency and childhood mortality has also been reported. In 2002, the United Nations had held a special session to examine ways to eliminate this problem through various approaches including breast-feeding, food fortification, improved diet and supplementation. Periodic supplementation of a mega dose of vitamin A has been determined to be suitable intervention at the community level, which is likely to improve compliance and cost effectiveness. This approach has resulted in reduction in all-cause deaths among under-five children by 23% in areas where vitamin A deficiency is a major public health problem [2] (<http://www.who.int/vaccines/en/vitamina.shtml> accessed on 26 May, 2005 at 1300 hrs). In view of the public health importance of vitamin A nutriture, the Government of Bangladesh recommends the administration of 200,000 IU of vitamin A every six months to all children in the age group of 12-59 months irrespective of their nutritional-and vitamin A status (Institute of Public Health Nutrition. Field guide for National Vitamin A Plus Campaign, 2005).

Vitamin A is required for maintenance of integrity of epithelial cells and its growth and differentiation, visual function, and the immune system [3]. Vitamin A deficiency leads to patchy keratinization of epithelial lining of the respiratory, genitourinary and gastrointestinal tracts [4], which acts as a protective barrier against infections. The loss of intestinal epithelial integrity increases the likelihood of bacterial colonization and incidence of infections and septicemia, which is strongly influenced by facilitating the passage of pathogenic bacteria into the systemic circulation [5] [6], [7] [8] {D.I.Thurnham, 2000 #99}. The vitamin A deficiency state is worsened during acute infections that reduces the dietary intake and absorption, and increases excretion of vitamin A in urine [9]. The mortality in vitamin A deficiency is also related to reduced immune function [10]; [11]. Vitamin A improves wound healing by mediating early inflammatory responses associated with influx of macrophages, enhanced fibroblast differentiation, and collagen accumulation [12].

About half of the absorbed vitamin A is oxidized and excreted in the feces and urine, and the remainder is stored in the liver as retinol ester. It is released into the plasma for transportation to peripheral tissues after binding with specific protein known as retinol binding protein (RBP). Protein deficiency in severe malnutrition reduces the hepatic synthesis of RBP [13], [14], which is the likely reason for low serum retinol level observed in such children [15-17]. Infectious diseases such as diarrhoea, and acute lower respiratory infections including pneumonia are common in malnourished children, which worsen the vitamin A deficiency state [18]. Acute infections are also associated with acute phase response and a transient decrease in serum retinol level [19]. This is due to reduced transcription of messenger RNA for RBP, resulting in decreased release of RBP from the liver into the blood [20] [21]. It is likely that liver utilizes all of its available resources for rapid synthesis of proteins that are required for the host defense in acute infection, thereby limiting the synthesis of less important ones such as RBP. In severe infection the increased urinary loss of retinol contributes to the lower serum retinol concentration [22]. Thus the utilization of vitamin A in the peripheral tissue is adversely affected in systemic infections and protein- energy malnutrition leading to development of features of vitamin A deficiency.

The two most common diseases that affect the under-five children are acute lower respiratory infections (including pneumonia) and diarrhoea. Earlier studies have shown the beneficial role of vitamin A as an adjunct therapy in the treatment of both watery and invasive diarrhoea [22, 23]. It was observed that an increase of 1 μmol/liter of retinol reduces proportion of patients with diarrhoea and respiratory disease by 50% and 80% respectively [8]. A more recent study had observed supplementation of vitamin A to under-five children to be associated with decreased frequency of diarrhoea, ALRI and subsequent malnutrition [24]. In another study 85% of the infants attending a diarrhoea treatment centre in Bangladesh were found to have depleted vitamin A stores, as measured by relative dose response test, and 61% of them remained deficient despite receiving high dose of vitamin A [22, 25]. Hospitalized Bangladeshi children receiving high dose of vitamin A experienced higher incidences of adverse manifestations such as nausea, vomiting and stool output [23] as well as higher incidence of severe nosocomial diarrhoea, while low dose vitamin A was associated with reduced incidence of severe diarrhoea in malnourished children [13]. In India, a study examined the effect of an intermediate dose (8.7 μmol; 2500 μgm), administered weekly, and did not find any difference in the incidence, severity and or duration of diarrhea in vitamin A deficient children [26]. Therefore, 2 vitamin A intervention studies in Indian infants revealed the results of Lactose:Mannitol (L:M) ratio which is the marker of impaired gut integrity to facilitates bacterial translocation and high L:M also indicates increased susceptibility to infection. One in a community based study, infants (aged 2-15 months) received 16700 IU vitamin A or placebo weekly for 8 weeks showed (L:M) ratio significantly improved at both 4 and 8 weeks after comparing with the base line values. However, no significant changes occurred in the placebo treated group. Another in a hospital based study, higher L: M ratio in high dose vitamin A supplemented group 30 days after discharge due to disruption of gut integrity in the hospitalized infants than community. The results suggesting impaired gut permeability was abnormal in the convalescence period of infants. Although, in presence of infection, release of vitamin A into the circulation will be hampered. In malnutrition, along with low plasma retinol concentration may delayed the repair of gut integrity. Therefore the Indian data is suggesting that intervantion with regular vitamin A supplements accelerated the gut integrity improvement in hospitalized children.

There are conflicting reports on the role of vitamin A supplementation in the management of pneumonia and acute lower respiratory tract infections [27]; [13]. Children with even sub-clinical vitamin A deficiency are at increased risk of developing ARI [28], [29]. Meta-analysis of community-based studies of vitamin A supplementation did not find either a protective or a detrimental effect of vitamin A supplementation on pneumonia-specific mortality in young children aged 6 months to 5 years (The Vitamin A and pneumonia working Group [30]. Some studies have actually reported an increase in the incidence of ALRI [31]. In a placebo controlled study, vitamin A supplementation did not influence recovery or duration of hospitalization in Guatemalan children with ALRI. [27]. A higher dose of vitamin A was evaluated in studies in Brazil [32], Vietnam [33], Chile [34] and Tanzania [35], and none of them observed any beneficial effect but suggested detrimental effect of vitamin A supplementation in adequately nourished children.. A subsequent clinical trial conducted in Peru not only observed vitamin A supplements to be ineffective in improving recovery from pneumonia in hospitalized children, but also observed prolonged duration of the clinical signs, higher requirements of oxygen support, and higher nursing time and patient care cost between the placebo group and high dose supplementation group where nutritional status did not influence the vitamin A status [36]. On the other hand, in a population based, controlled clinical trial in Ecuadorian urban slum children, weekly supplementation of low dose vitamin A (10,000 IU) for 40 weeks was associated with a strong protective effect on ALRI in malnourished under-five children [37]. A study of randomized trial of vitamin A supplements among HIV infected children in Tanzania reported that severity of pneumonia may detoriate when high dose of vitamin A given during acute episodes. Therefore different effect of vitamin A depends on the nutritional vitamin A status of the children.

The reports on mortality and morbidity of diarrhoea and pneumonia in severely malnourished children associated with vitamin A supplementation are also conflicting. Some studies have observed no significant difference in mortality (8% vs. 8.4%) or in hospital stay (10 days vs. 9 days) in high and low dose groups respectively [13]. However, in sub-group analysis significantly lower deaths was observed among oedematous children who received low dose vitamin A on admission [38].

A meta-analysis observed substantial reduction in mortality in association with vitamin A supplementation in population where at least low-level deficiency state was prevalent, and the effect was independent of dose, sex, and age, and the effect was more pronounced for incidence of diarrhoea than for respiratory diseases [39]. However, no difference in morbidity (frequency, duration, or prevalence) from diarrhoeal or respiratory diseases was noted (Ghana VAST Study Team) [40] among stunted children in Ghana.

Malnourished children are deficient in retinol binding protein (RBP) and thus the resultant free, unbound form of vitamin A is excreted either through urine or faces, and the situation is aggravated in acute infections. Vitamin A, when administered in a single high dose, presumably circulates in free form in the extra cellular fluids before being taken up by the liver. This free form of vitamin A perhaps affects sodium and water metabolism causing ‘pseudotumor cerebri’, an adverse event of hypervitaminosis A [41]. Lack of adequate food is an important cause of malnutrition, and the bioavailability of vitamin A from the consumed plant and meat source is also low **(**Dietary Reference Intakes for Vitamin A. <http://www.nap.edu/openbook/0309072794/html/ 6.5.html. Accessed> May 20, 2005 at 1400 hrs). A national nutritional survey in Bangladesh [42] observed vitamin A deficiency as a significant public health problem in various population groups. Although the prevalence of clinical signs of vitamin A deficiency greatly reduced from 3.6% in 1982-83 to 0.6% in 1996 in Bangladesh, the prevalence of sub-clinical vitamin A deficiency among the preschool children remained higher in a hospital based study with small sample size [42].

**Rationale**

Vitamin A supplementation has been proven to be beneficial in the management of clinical and sub-clinical deficiencies of this important micronutrient. Such supplementation has also been found to improve outcomes from certain illnesses, particularly diarrhoeal diseases and also in preventing infectious diseases. The beneficial effect of vitamin A supplementation in acute lower respiratory infections (and pneumonia) is not that clear, however. WHO currently recommends single high dose of vitamin A supplementation for malnourished children with diarrhoea because this programme is relatively low cost and rate of participation is high for improving health status of children. The WHO recommendation is based on studies in communities that showed benefit of the cheapest intervention, which is quicker to implement into health programmes. The programmes for vitamin A capsule distribution reduced risk of vitamin A deficiency in children who participated in this program. Interpretation of the three studies done in the Democratic Republic of Congo and Senegal, as mentioned earlier, in relation to the existing guidelines on vitamin A supplementation was considered difficult and their validity was doubted in a meeting convened by WHO in September 2004. It was, however, accepted that the question of vitamin A dosage is an important one and there is scope for further research. Repeated dosing needs to be approached cautiously for HIV-symptomatic and other children who are continually being readmitted, and the meeting proposed that the high dose only be given if the interval is at least 6 months from the last previous high dose. Tissue level of vitamin A/other functional consequences of vitamin A deficiency may not be effectively controlled in malnutrition [43]. No effect on morbidity from diarrhoea has been observed with high dose vitamin A whereas in several trials mortality was reduced with low dose vitamin A and the impact of daily low dose vitamin A on diarrhoea and respiratory tract infections of malnourished children is poorly studied.

In Bangladesh, high potency (200,000 IU) vitamin A is routinely supplemented to under-five children every 6 months. Malnourished children lack in serum retinol binding protein, which is synthesized in the liver and is required for carrying retinol to the target organs and tissues. Malnutrition is also associated with reduced absorption of vitamin A precursors from the intestine. Therefore, it can be assumed that a significant proportion of supplemented vitamin A is excreted in faces and urine, and urinary excretion is substantially increased in acute infections including diarrhoeal diseases that malnourished children frequently suffer from. On the other hand, free vitamin A, a consequence of reduced RBP, increases their concentration in the body and thereby might increase the possibility of adverse events such as “pseudotumor cerebri”. Therefore, there may not be any benefit from supplementation of initial high dose to severely malnourished children. There are at least three large studies that failed to observe any effect of mega dose of vitamin A on the incidences and duration of diarrhoea and respiratory tract infections. In recent studies it has been shown that daily low-dose supplementation of vitamin A to malnourished under-five children produces similar or better effect, compared to high dose, in acute illness and also in preventing infectious diseases. There was higher mortality rate in high dose group compared to the low dose group but the study was done in a smaller number of participants [13, 38] which should be addressed in well-designed clinical trial(s) of high dose versus low dose vitamin A in severely malnourished children with adequate sample size. In serum, vitamin A remains saturated with RBP in a 1:1 ratio and, therefore, the amount of vitamin A in excess of this ratio, as would be expected in mega-dose supplementation of vitamin A to severely malnourished children with low serum RBP, would be non-functional at best [17]. Two trials in Asia that used high dose vitamin A observed conflicting results [44, 45]. On the other hand, weekly supplement of 8333 IU vitamin A was reported to reduce mortality by 54% [46]. There thus is a need for a carefully designed study with adequate sample size to address the effect of low dose vitamin A on morbidity and mortality among severely malnourished children with diarrhoea and pneumonia- the basis for our proposed study. We hope that the results of this study will enable us to compare the cure rates of low-dose daily administration of vitamin A with that of initial mega dose followed by daily low dose of vitamin A. Daily basal requirements (estimated amount which is needed to prevent signs of deficiency) for 0-1 and 2-6 years old children are 600 and 650 IU respectively; normal requirement (which is sufficient to maintain desirable levels of tissue store) for the same age group are 1200 and 1300 IU respectively [43]. It is estimated that malnourished children will lose 0.1µmol of retinol /day (97 IU) through urine during acute illness ([22].

The national coverage rate (percentage of target age children who received vitamin A capsules during the past six months, according to the mother) of vitamin A in rural areas was 45% in 1982-83[47]. The rate gradually decreased to 37% in 1987-88 and 35% in 1989 respectively [48] [42]. However the Nutritional Surveillance Project reported in December 1995, that the coverage of vitamin A capsule distribution increased substantially and it was 83.6% in rural areas and 73.7% in urban areas [42]. It is noteworthy that night blindness is considered as the marker of vitamin A deficiency, and when that exceeds 1%, is considered as a significant public health problem . In Bangladesh, proportion of children aged 18-59 months having night blindness in rural areas and urban slums were 0.23% and 0.12% respectively. These figures are well below the cut-off for public health significance of vitamin A deficiency. Anecdotal observations of doctors working at the ICDDR,B Hospital, Dhaka, indicate a significant decrease in the number of children with clinical vitamin A deficiency seeking treatment in the hospital. According to a report of the surveillance system of Dhaka Hospital of ICDDR,B 84 children presented with conjunctival xerosis and only three children with keratomalacia/corneal ulcer out of a total of 11605 under-5 children during the period 1996-2004. The children enrolled in our proposed study will receive 5000 IU of vitamin A, and additional 1000 IU during the acute phase and 800 IU during the nutritional rehabilitation phase of severe malnutrition from various diets provided to them during their hospital stay. Thus we estimate that the total amount of vitamin A received from these sources, even after adjusting for urinary excretion and the retinol level, will not fall below the basal and normal requirements. We feel that once we are able to maintain the daily optimal level of vitamin A store in the body the sudden development of keratomalacia can be averted. However, measures will be taken through clinical examination (of eye and skin) to exclude children with any form of clinical vitamin A deficiency at the time of enrollment. Children having clinical vitamin A deficiency disease (conjunctival xerosis, Bitot’s spots, keratomalacia, or dermatosis suggestive of vitamin A deficiency) will not be eligible for enrollment and will be provided with the currently recommended treatment with vitamin A supplementation as is routinely followed in this hospital. It may also be noted that a couple of studies did not observe development of an overt sign of vitamin A deficiency in children not receiving vitamin A (placebo group) [13, 36]. Additionally, serum retinol concentrations were not significantly changed after 7 days of hospitalization both among children receiving the high dose and children receiving the low dose or placebo [13].

**Research Design and Methods**

Describe in detail the methods and procedures that will be used to accomplish the objectives and specific aims of the project. Discuss the alternative methods that are available and justify the use of the method proposed in the study. Justify the scientific validity of the methodological approach (biomedical, social, or environmental) as an investigation tool to achieve the specific aims. Discuss the limitations and difficulties of the proposed procedures and sufficiently justify the use of them. Discuss the ethical issues related to biomedical and social research for employing special procedures, such as invasive procedures in sick children, use of isotopes or any other hazardous materials, or social questionnaires relating to individual privacy. Point out safety procedures to be observed for protection of individuals during any situations or materials that may be injurious to human health. The methodology section should be sufficiently descriptive to allow the reviewers to make valid and unambiguous assessment of the project. **(DO NOT EXCEED TEN PAGES, USE CONTINUATION SHEETS).**

**Study design**

This will be a randomized, double blind, controlled clinical trial examining the effects of daily low dose versus single large dose followed by daily low dose of vitamin A supplementation on the diarrhoea and ALRI/pneumonia morbidities of severely malnourished hospitalized children.

Study site

The study will be conducted at the Dhaka hospital of ICDDR, B. The hospital is located in Dhaka, the capital city of Bangladesh that has a population of over 13 million. Each year, around 100,000 diarrhoeal patients, with and without complications, attend the hospital seeking treatment for their illnesses, and about 6000 of them, mostly under-five children, require admission to the longer stay wards for complications of diarrhoea or presence of other infectious diseases, particularly ALRI/pneumonia and sepsis, and severe malnutrition (45 to 50%).

Inclusion criteria

1. Age 6-59 months
2. Either sex
3. Severe malnutrition as defined by the presence of any of the followings:
   1. Bipedal oedema
   2. Weight (measured after correction of dehydration) for height Z score <-3 of the National Center for Health Statistics (NCHS) reference
   3. Written informed consent of respective parents/ guardians for participation of the children in the study.

Exclusion criteria

1. Failure to obtain consent
2. Received a dose of vitamin A within the last three months
3. History of night blindness or eye signs of vitamin A deficiency
4. Measles or history of measles within last 8 weeks [49]
5. Clinical suspicion of TB (evening rise of temperature, loss of appetite, gradual loss of weight, cough, night sweating) after applying modified Kenneth Jones criteria [50], shock other than due to hypovolaemia or hypoglycaemia, congestive cardiac failure, severe sepsis (hypothermia, tachycardia, tachypnea, hypotension) [51], and seizures disorders
6. Trisomy-21
7. Cerebral palsy
8. Any other known chronic disease (eg. hepatic, renal or congenital disorder) or malignant condition.

Final inclusion in the study

Dehydration would be assessed using a modified WHO guidelines (Dhaka Method)[52] that is routinely practiced at the hospital. Dehydration would be managed using oral or intravenous fluids, as appropriate, following the WHO guidelines. A final diagnosis of acute infection, either diarrhoea or ALRI/pneumonia or both, will be made after the correction of dehydration and patient will be enrolled in the study.

Study Interventions

There will be two treatment groups: (i) test intervention, and (ii) comparator.

1. Treatment group: Will receive four capsules of vitamin A placebo orally (2 capsules for children aged <12 months) on the day of enrollment plus an oral dose of 5000 IU of vitamin A (in the form of multivitamin drops), and the same oral dose of vitamin A for the next 14 days i.e. throughout their entire nutrition rehabilitation phase in the hospital.
2. Comparator group: Will receive oral mega dose (4 capsules of vitamin A, each containing 50,000 IU i.e. a total of 200,000 IU; 2 capsules for children aged <12 months i.e. a total of 100,000 IU) plus an oral dose of 5000 IU of vitamin A (in the form of multivitamin drops), and the same oral dose of vitamin A for the next 14 days i.e. throughout their entire nutrition rehabilitation phase in the hospital.

A local pharmacutical company (Drug International, Bangladesh) will prepare and supply vitamin A capsules and placebo for the study. One of the investigators will administer the study interventions to the participating children following the randomization schedule at a fixed time of each day, and the date and time of actual administration will be recorded. Randomization will be done by an experienced scientist, not involved in the study in any way, using a block randomization method with variable sizes of blocks between 2 to 6.

Sample size

The sample size of this study has been estimated on expected proportions of clinical success in two groups. Earlier studies have observed 69% and 65% clinical recovery among children suffering from shigellosis and cholera respectively [53, 54], and 60% for pneumonia (personal communication with Dr Dolly Goswami, Kamlapur field site of ICDDR,B) within 48 hours when treated with an effective antimicrobial agent. We assume that the recovery rate would remain same (i.e. 70%) in the high followed by low dose vitamin A group and there would be 15% increase in the rate i.e. 85% in the low dose vitamin A group. Considering 80% power at 5% significance the sample size is estimated as follows:

N = P_1_ (100-P_1_) + P_2_ (100-P_2_) × F ( αβ ) where P1= 70% and P2 = 85%.

(P_1_- P_2_)^2^

= 118. αβ = 7.84

Thus the required sample size is 118 in each group, and with an anticipated dropout rate of around 10% the sample size will be 130 children in each of the two treatment groups i.e. a total of 260 children.

Patient recruitment, Clinical and Laboratory Assessments

1. **Patient recruitment**
   1. Children will be screened for recruitment between 9 am to 4 pm everyday. A trained research assistant will initially screen them. After explaining the study including its aims, procedures, interventions and their possible risks and benefits, rights to refuse participation and withdrawal of consent during study, and confidential handling of information the parents/guardians would require to give consent to enroll their children. The same research assistant will then perform anthropometric measurements including height, weight and mid upper arm circumference, and calculate weight for age, height for age and weight for height percentiles taking National Center for Health Statistics (NCHS) median as the standard. Z-scores will be computed and children with weight for height Z-score less than 3 will be enrolled in the study .
   2. Final enrollment: After rehydration, children will be re-weighed, and final enrollment will be done if weight for height Z-score less than 3 .
2. **Clinical Assessments**
   1. Research assistant will collect the demographic and other data using a pre-designed clinical record form (CRF).
   2. One of the investigators will obtain pertinent medical history and perform through physical examination, and also ensure that the children meet eligibility criteria. All information would be recorded on a pre-designed and pre-tested case report form (CRF).
   3. Randomization: Patient will be randomly assigned to either of the two interventions based on a computer generated randomization table.
   4. During hospitalization data will be collected on the outcome variables as well as relevant clinical information including type and severity of diarrhoea and pneumonia.
   5. All the patients will receive standard treatment as per the hospital’s guidelines.
3. Laboratory assays
   1. **Blood test**s: Blood for retinol and RBP, glucose, complete blood count, serum electrolytes, total protein, albumin and, as measures of infection status, pre-albumin and C-reactive protein (CRP) will be measured after randomization on the admission day. Serum retinol and RBP will also be measured on study day-3 and 15. Any other heamatological test will be repeated if only clinically indicated. . Approximately 3.0 ml of venous blood will be adequate to perform all of the tests.
   2. Microscopic examination of stool: Stool microscopic examination for leukocyte and erythrocytes, and identification of pathogens such as *Cryptosporidium, Giardia lamblia* and *E. histolytica*.
   3. Stool or rectal swab culture: For isolation of *Shigella*, *Salmonella*, *Vibrio cholerae* on admission, and at other time(s) only if clinically indicated.
   4. Stool ELISA: Would be done for identification of rotavirus on admission.
   5. Urine M/E: Urine microscopic examination for cells, casts, pH, protein and sugar. Urine culture may be performed when clinically indicated.
   6. X-ray chest: To identify abnormalities if any on admission, and at other time(s) only if clinically indicated.
   7. Pulse oximetry: Will be done for children with ALRI/pneumonia on admission, and at least once daily until oxygen saturation is stable at 99-100% on two consecutive days.
4. Laboratory methods

Serum retinol will be measured by high-pressure liquid chromatography (HPLC) method adapted from the method of Vanderpas and Vertongen [13, 55]. RBP will be measured with a single-radial immunodiffusion technique using commercially available plates (The Binding site, Birmingham, United Kingdom). Ring diameters (in mm) will be read with an electronic single-radial immunodiffusion plate reader (The Binding ite, Birmingham, United Kingdom) 72 hours after inoculation of serum sample (the precipitation ring will be hazy, large or small to read). For each plate, 3 wells will be used for standards of different concentrations and 1 for quality control[22].

Serum retinol concentration and grading of vitamin A deficiency will be done according to WHO criteria, as follows:

Retinol conctration μmol/l (μg/dl): Grading of Vitamin A deficiency

< 0.35 (< 10) : Deficient

0.35 to 0.70 (10 to20) : Low

 0.70 (> 20) : Normal

1. Daily examination and recordings

One of the investigators will assess study children at least once daily, usually in the morning; however, additional assessments may be performed in the interim if deemed necessary for clinical management. Each child will be weighed every morning, without clothes, to the nearest 0.5 gm using an electronic weighing scale (model 6810; Seca Corporation, Columbia, MD). Height, or recumbent length in children less than 2 years of age, will be measured with a standard height or length board to the nearest 0.1centimeter. MUAC will be measured midpoint between the elbow and the shoulder of the left arm with a non-expanding tape and recorded to the nearest 0.1 cm. Rectal temperatures will be measured 3 times/ day.

1. Morbidity evaluation

Morbidity data such as presence of cough, difficult breathing, fever and other symptoms, and findings of physical examination including pulse and respiratory rates, presence of oedema, mental status (lethargy, irritable, drowsy, obtundation, coma), dehydration status, lower chest wall in-drawing will be recorded. Detailed inquiries will be made about stool frequency, consistency and contents (mucus, blood, undigested food etc).

1. **Discharge from the hospital**: Patients will be discharged after recovery.
2. Definition of recovery from acute phase illness

**Clinical success:**

**Diarrhoea:** Will be defined as cessation of watery/abnormally loose stool, and disappearance of visible blood (for children with dysentery) within 48 hours of starting of an effective therapy.

**Pneumonia**: will be defined as disappearance of clinical signs (i.e. crepitations if present) and symptoms of pneumonia/ALRI within 48 hours of starting an effective therapy

1. **Case Definitions**

**Diarrhoea**: Diarrhoea is defined as the passing of liquid or watery stools more than 3 times in previous 24 hours [56].

**Stool consistency:** Stool consistency will be categorized as follows:

1. Watery: That can be poured from one container to another container easily. Like water with no/little attachment to the container.

2. Soft: That cannot be poured from one container to another container easily, like water, but quickly takes the shape of the container.

3. Formed: That retains its shape.

1. **Assessment of dehydration**

Dehydration will be assessed in accordance with the modified WHO guideline as used at the Dhaka Hospital of ICDDR, B[57].

1. **Acute lower respiratory tract infection (ALRI) / Pneumonia: [58]**

In the proposed study, pneumonia would be defined according to the following criteria.

A child presenting with cough or difficult breathing with any of the following:

1. Fast breathing: This will be defined according to age groups.
   1. 6 – 11 months = > 50 breaths per minute
   2. 12 – 59 months = > 40 breaths per minute
2. Crackles on chest auscultation
3. **Grading of ALRI [13]**

- ALRI1- defined as cough and increased respiratory rate (according to age) of the patients.
- ALRI2 - defined as cough and rectal temperature of 38.5^0^C or more at least once in a 24-hour period.

1. **Sign of recovery from pneumonia [58]**

Within 48 hours-sign of improvement: Specific respiratory rate according to age will be decreased.

1. **Severe pneumonia [58]: Will be diagnosed based on the presence of the followings:**
2. Cough
3. Lower chest wall indrawing (the lower part of the chest moves in or retracts when inhalation occurs in a calm child).
4. Fever (may be absent)
5. Lungs auscultation: crackles, and decreased or bronchial breath sounds.
6. **Case Management**

**Management of severe malnutrition:** Would be done according to the protocolized management of severely malnourished children at Dhaka hospital [57]. This protocol is based on WHO guidelines for management of severe malnutrition[59]

**Management of dehydration:** Dehydration will be managed following WHO criteria in malnourished children [57](<http://w3.whosea.org/techinfo/pdf/nutrmanu.pdf> accessed on 12 July,2005 at 1000 hrs). For children with severe dehydration (body weight loss of 10% or greater) initial hydration will be done with intravenous polyelectrolyte solution (“Cholera saline” containing sodium, potassium, chloride and bicarbonate of 133, 13, 98 and 48 mmol/L respectively) in an amount of 20ml/kg in the first hour and 10ml/kg in the second hour; the rest of the estimated deficit (70ml/kg) will be corrected using ORS solution (sodium, potassium, chloride, citrate and glucose [or rice powder] of 75, 20, 65, 10, and 75 [rice powder 40 gm/L] respectively). Calculated total intravenous fluid will be administered as drops per minute (1ml = 60 drops by a ‘micro drip’ or a ‘soluset’). ORS solution would be administered @10ml/kg each hour during the initial 2 hours and then @ 5 ml/kg each hour until full hydration is achieved. The ongoing stool loss will be replaced by administering 5-10 ml/kg of ORS for each watery stool. In the event children are unable to drink ORS solution due to any reason (extreme lethargy, frequent vomiting, and unconsciousness) that would be administered through a NG tube.

**Antimicrobial therapy for infectious illness**

The standard antimicrobial therapy for management of severely malnourished children at the Dhaka hospital of ICDDR,B will be used for management of the study children.

Pneumonia: children will receive parenteral chloramphenicol in a dose of 75-100 mg/kg.day in 4 equally divided doses for the first 2 days followed by an oral formulation in the same dosage for the next 5 days.

If no clinical improvement is observed to occur within 48 hours (i.e. patients remain febrile and or respiratory rate does not come down) or if the clinical condition deteriorates after 24 hours of treatment, chloramphenicol will be replaced by parenteral ceftriaxone to be administered in a dose of 100mg/kg once daily plus gentamicin 1.5 mg/kg parenterally every 8 hours for 7 to 10 days.

Diarrhoea: Children with cholera will be treated with erythromycin administered in a dose of 12.5 mg/kg body weight every 6 hours for 3 days, and those with shigellosis will be treated with 12.5 mg/kg of pivmecillinan every 6 hours for five days.

**Micronutrients**

The following vitamins and minerals are routinely provided to severely malnourished children admitted to the Dhaka hospital of ICDDR,B: elemental zinc and folic acid are administered in a dose of 2 mg/kg and 1.25 mg/day respectively for 15 days; magnesium sulfate is administered in a dose of 0.4 mmol/kg as an intramuscular injection once a day for seven days. Multivitamin (MV) drops are provided in a dose of 1 ml daily. One millilitre of commercially available MV drops contains vitamin A (as palmitate) 5,000 IU, vitamin D 1,000 IU, thiamine hydrochloride 1.6 mg, riboflavin 1 mg, pyridoxine hydrochloride 1 mg, nicotinamide 10 mg, calcium D-pantothenate 5 mg, and ascorbic acid 50 mg.

**Management of associated conditions**

1. Hypoglycemia (blood glucose <3mmol/L or 54mg/L, measured by Dextrostix): Patients with symptomatic hypoglycemia, presenting with unconsciousness or convulsions, would be given 2ml/kg of 25% glucose immediately through intravenous route followed by 10% glucose orally or by nasogastric tube (for patients who are unable to drink due to reasons such as unconsciousness, weakness or frequent vomiting) for prevention or recurrence of hypoglycemia.
2. Hypokalaemia (serum potassium of < 3.5mmol/L) [60]: Manifestations of hypokalaemia are usually evident with a serum potassium concentration of 2.5mEq/L or below [61]. Children may require intravenous infusion due to severe dehydration. In that case, if serum potassium is <2.0 mmol/L potassium chloride will be added to infusion to raise the potassium concentration to 40 mmol/L, and if serum potassium is between 2 to 2.5 mmol/L, potassium in the infusion solution would be increased to 30mmol/L. Oral potassium chloride, 4 mmol/kg.day (5 ml syrup containing 7 mmol of elemental potassium), will be supplemented 3 times/day for 5 days to children without manifestation of hypokalaemia.
3. Thrush (oral candidiasis): Nystatin oral drops 1 ml (100,000 units) will be given 4 times daily for 5 days or until cure.
4. Specific infections: Will be treated with appropriate antimicrobial, as determined by susceptibility report.
5. Complications: During acute phase treatment, complications will be managed according to cause.
6. Diet: For children presenting with dehydration, feeding will be started as soon as rehydration is accomplished or as soon as possible after admission. Diet would be given in the form of 10 ml/ kg every 2 hour of milk suji, which provides 67 kcal/100ml of energy, by nasogastric tube (to ensure intake by patients with poor appetite, repeated vomiting, weakness and painful oral lesion e.g. stomatitis/glossitis/gingivitis) until they are able to resume oral feeding. In addition, semisolid foods and milk suji 100 (high energy milk-based therapeutic diet) would be given to the children when they enter the nutritional rehabilitation phase. The management of this phase is based on a standardized dietary protocol which is routinely followed in the Nutritional Rehabilitation Unit (NRU) of the hospital.
7. **Outcome variables**

**Primary outcome**

1. Resolution of diarrhoea
2. Resolution of ALRI

**Secondary outcomes**

1. Duration of acute phase of illness
2. Time to nutritional rehabilitation (achieving of 80% of W/H)
3. Rates of weight gain
4. Morbidity developed during hospitalization such as nosocomial infections
5. Serum retinol and RBP on admission and on day 15
6. Case fatality rates

**Data Analysis**

Describe plans for data analysis. Indicate whether data will be analyzed by the investigators themselves or by other professionals. Specify what statistical software packages will be used and if the study is blinded, when the code will be opened. For clinical trials, indicate if interim data analysis will be required to monitor further progress of the study. **(TYPE WITHIN THE PROVIDED SPACE).**

**Data Analysis:**

Data will be entered onto personal computer using SPSS statistical package and statistical analyses will be performed using the same program, and z- scores will be determined using Epi Info 2001. Normally distributed continuous variables will be compared by t-test for normally distributed data and Mann Whitney U test will be used for data that are not normally distributed. Time to recovery would be compared by log rank test (Kaplan Mayer survival analysis). Two by two tables will be constructed to relate the findings of discrete variable such as chest X-ray findings of pneumonia (present or absent) and Fisher’s exact test would be performed where indicated. Statistical significance will be set at 5% level.

**Ethical Assurance for Protection of Human Rights**

Describe in the space provided the justifications for conducting this research in human subjects. If the study needs observations on sick individuals, provide sufficient reasons for using them. Indicate how subject’s rights are protected and if there is any benefit or risk to each subject of the study.

**Justifications for conducting this research in human subjects**

Vitamin A deficiency is a major nutritional problem in developing countries including Bangladesh, and under-five children, particularly malnourished children are at higher risk. ALRI/pneumonia and diarrhoea are the two most common illnesses of under-five children. The beneficial role of high dose vitamin A supplementation, as an adjunct therapy in the treatment of diarrhea, has been documented in different studies. However, high dose vitamin A supplementation did not produce any beneficial effect on recovery from ALRI in well-nourished children with adequate stores, rather it increased the incidence of ALRI. WHO estimated that the lives of one million children could be saved globally each year through high dose supplementation of vitamin A. The results of five large trials in Asia have observed 20-30% drops in deaths from diarrhoeal diseases and pneumonia, and about one-third reduction in clinic attendance rate, hospital admissions and morbidity among vitamin A supplemented children. However, other studies have observed higher incidences of adverse events among malnourished children receiving high dose of vitamin A supplementation. The proposed study will examine if daily administration of a low dose of vitamin A produces similar effect on disease morbidity and lesser incidence of adverse events as compared to children receiving high dose vitamin A followed by daily administration of a low dose of vitamin A.

##### Protection of human rights

The intent of the research program, the study protocol, and the informed consent form to be used in the study will be submitted to the Ethical Review Committee of ICDDR, B and the study would be initiated only after receiving approval of the Committee.

**Informed Consent**

Written informed consent will be obtained from parent/guardians of each patient, in presence of a witness, before they can be enrolled in the study. It would be the responsibility of the investigators to obtain valid written informed consent from the parent/guardian after adequate explanation of the aims, methods, anticipated benefits and potential hazards of the study. The investigator must also explain to the parents/guardians that they are completely free to refuse to participation of their children in the study and also withdraw them from the study at any time during the study without showing any reason and without affecting their further treatment at the hospital for the current illness as well as in future. Parents/guardians will indicate their consent by signing the consent form or by putting their left thumb impressions (those who are unable to write), along with the date.

**Literature Cited**

Identify all cited references to published literature in the text by number in parentheses. List all cited references sequentially as they appear in the text. For unpublished references, provide complete information in the text and do not include them in the list of Literature Cited. There is no page limit for this section, however exercise judgment in assessing the “standard” length.

**Dissemination and Use of Findings**

Describe explicitly the plans for disseminating the accomplished results. Describe what type of publication is anticipated: working papers, internal (institutional) publication, international publications, international conferences and agencies, workshops etc. Mention if the project is linked to the Government of Bangladesh through a training programme.

The findings of the study will disseminated as follows:

1. Presentation(s) at Centre’s Scientific Forum (CSF), ICDDR, B for dissemination amongst Centre scientists.
2. Publication in peer-reviewed international medical journal.

**Collaborative Arrangements**

Describe briefly if this study involves any scientific, administrative, fiscal, or programmatic arrangements with other national or international organizations or individuals. Indicate the nature and extent of collaboration and include a letter of agreement between the applicant or his/her organization and the collaborating organization. **(DO NOT EXCEED ONE PAGE)**

This study will be conducted in collaboration with Khulna Medical College.

**APPENDIX**

**International Centre for Diarrhoeal Disease Research, Bangladesh**

###### Voluntary Consent Form

**Title of the Research Project:** The effect of 2,00,000 IU of vitamin A followed by 5,000 IU daily dose versus daily 5,000 IU of vitamin A on recovery from diarrhoea and acute lower respiratory infections in severely malnourished hospitalized children.

Principal Investigator: Dr. Shamima Sattar/ Dr Md Iqbal Hossain

Before recruiting into the study, the study subject must be informed about the objectives, procedures, and potential benefits and risks involved in the study. Details of all procedures must be provided including their risks, utility, duration, frequencies, and severity. All questions of the subject must be answered to his/ her satisfaction, indicating that the participation is purely voluntary. For children, consents must be obtained from their parents or legal guardians. The subject must indicate his/ her acceptance of participation by signing or thumb printing on this form.

**Introduction**

Vitamin A deficiency is a major nutritional problem, which is common in under-5 age group, particularly in malnourished in developing countries, including Bangladesh. Malnourished children more frequently suffer from diarrhoeal diseases and pneumonia. Thirty percent of the malnourished children admitted at ICDDR,B, Dhaka hospital are under the age of 5 years.

**The study and its purpose**

Your child is suffering from malnutrition in addition to diarrhoea and/or pneumonia. It has been observed in earlier research studies that children like yours are also deficient in vitamin A. In this hospital, 200000 IU of vitamin A is routinely given to all malnourished children on the day of admission, followed by 5000 IU of vitamin A in multivitamin drops till recovery from severe malnutrition. It has been observed that high dose vitamin A does not help in reducing morbidity and in some instance it might increase the risk of death. Results of other research studies indicate that if vitamin A is given in low dose for a longer period, that helps in early recovery from childhood disease e.g. diarrhoea, pneumonia, and also reduces deaths among them. We are planning to conduct a study to see the effect of daily low dose (5000 IU) with that of a single high dose (2000,000 IU) followed by daily low dose of vitamin A given for 15 days in severely malnourished children with either diarrhoea or pneumonia or both. Since your child is malnourished and she/he has pneumonia / diarrhoea and likely to be vitamin A deficient, we intend to enroll your child in the study.

**Methods**

If you agree to our proposal for inclusion of your child in this study, the following would be done:

- - 1. We will admit your child in this hospital for at least 15 days. .
    2. She/he will receive the usual good care of this hospital.
    3. We will ask questions related to her/his illness and diets, and perform thorough physical examinations on admission and on each day in this hospital, with particular attention to nutritional disorders, dehydration, and other associated health problems.

4. We would collect 5.0 ml (about one teaspoonful) blood on the day of admission and another 1.0 ml (1/5^th^ of a teaspoonful) of blood on 15^th^ day of hospitalization for various laboratory tests. We would collect a small amount of stool for various tests, and a rectal swab specimen, and perform her/his chest x-ray on admission day. These tests or other tests might also be required at other times for assessment of her/his condition and determine treatment.

5. By chance alone, your child will receive either 4 capsules of vitamin A, each containing 50,000 IU (2 such capsules if aged <12 months) plus 1.0 ml of multivitamin drop containing 5000 IU of vitamin A in multivitamin daily for 15 days. Neither you nor the investigators of this study will know which of the two is study will know involved in the study will not know what group of medication your child is getting.

**Your rights**

Participation of your child in this study is entirely voluntary. You may decide for or against participation of your child in this study. You would also be able to may also withdraw your child at any time during the study. Your child would receive the usual good care and treatment of this hospital if she/he does not participate in the study and also if you withdraw her/him from the study before its completion.

**Risks**

There is no risk to your child / relative from physical examination, and she/he will feel momentary pain during insertion a needle for collection of blood from a vein on the forearm. There is a small chance of bluish discoloration of the surrounding skin around the needle prick due to oozing of blood from the puncture site, and remote possibility of local and systemic infections. We would use sterile, disposable syringe and needle for collection of blood and also take aseptic precaution to prevent these.

**Benefits**

Your child may or may not benefit from participation in this study; however, results of this study might be helpful in improving management of malnourished children in future.

**Confidentiality**

All information of your child collected from you and results of his / her laboratory tests would be kept confidentially in a secured place. None other than the investigators involved in the study and the members of the Ethical Review committee would have an access to that information. We would provide you with the laboratory result, when they become available. However, some of the results will be available only after the completion of the study. The matter of confidentiality and study disclosure will be in accordance with the law of the country. The name or identity of your child would not be used in the analysis of data or in publishing the results of this study.

**Right to ask questions**

We would be happy to answer your questions about the illness and the study now, you should be able to contact the principal investigator of this study personally in this hospital or over telephone at the following address.

Dr. Shamima Sattar, Clinical Sciences Division, ICDDR, B, Mohakhali, Dhaka 1212, Telephone No. 8811751 through 881160: Extension 2332.

Declaration by the parent / guardian:

If you agree to participate your child / relative in this study, please sign / put your left thumb impression at the specified space below:

__________________________ _________________ _____________________________________

**Signature of Subject/ Guardian Signature of witness Signature of Investigator/ or representative**

**Date: Date: Date:**
